# Supplementary material for: Genome Analysis of a Novel Polysaccharide-Degrading Bacterium Paenibacillus algicola and Determination of Alginate Lyases
Source: Mar Drugs. 2022 Jun 9;20(6):388. doi: 10.3390/md20060388 (PMC9227215; doi:10.3390/md20060388)
Supplement: Supplementary file 1 [file marinedrugs-20-00388-s001.zip › marinedrugs-1696187-supplementary.pdf]

marine drugs

MDPI

Article

Genome Analysis of a Novel Polysaccharide-Degrading Bacterium *Paenibacillus algicola* and Determination of Alginate Lyases

Huiqin Huang [1,2,3], Zhiguo Zheng [1,2,3], Xiaoxiao Zou [1,2,3], Zixu Wang [1,3], Rong Gao [1,4], Jun Zhu [1,2,3], Yonghua Hu [1,2,3,5*], Shixiang Bao [1,2,3*]

1. Institute of Tropical Bioscience and Biotechnology, Hainan Institute for Tropical Agricultural Resources, CATAS, Haikou 571101, China; huanghuiqin@itbb.org.cn (H.H); zhengzhiguo52@163.com (Z.Z.); zouxiaoxiao@itbb.org.cn (X.Z.); ev-er@hainanu.edu.cn (Z.W.); gryt2727@163.com (R.G.); zhujun@itbb.org.cn (J.Z.)
2. Zhanjiang Experimental Station, CATAS, Zhanjiang 524013, China
3. Hainan Provincial Key Laboratory for Functional Components Research and Utilization of Marine Bioresources, Haikou 571101, China
4. College of Oceanography, Hebei Agricultural University, Qinhuangdao 066000, China
5. Laboratory for Marine Biology and Biotechnology, Pilot National Laboratory for Marine Science and Technology, Qingdao 266071, China

Correspondence: huyonghua@itbb.org.cn (Y.H.); baoshixiang@itbb.org.cn (S.B.);
Tel: +86-898-66890671 (Y.H.); +86-898-66890671 (S.B.)

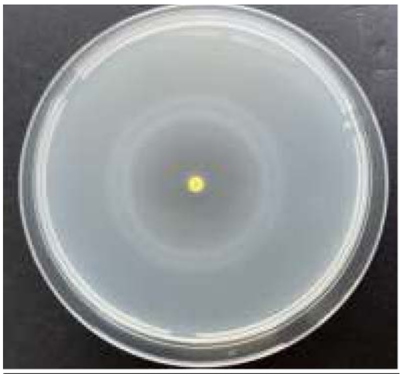

Figure S1. Gellation reactions of strain HB172198^T observed on the plate covered by $CaCl_2$ solution

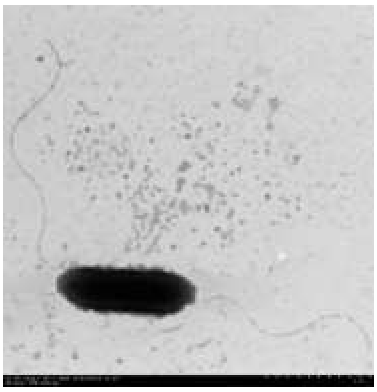

Figure S2. Transmission electron micrograph of cells of strain HB172198^T from a 2-day-old culture on MA. Bar, 2 μm.

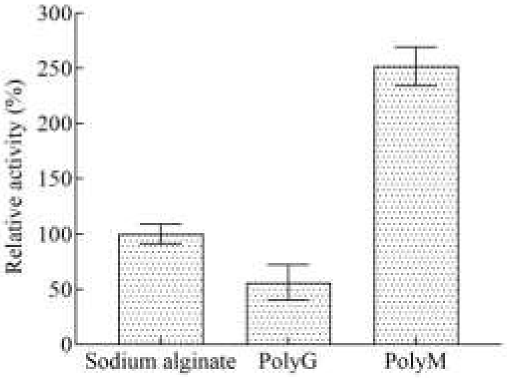

Figure S3. Substrate specificity of the alginate lyases from strain HB172198^T

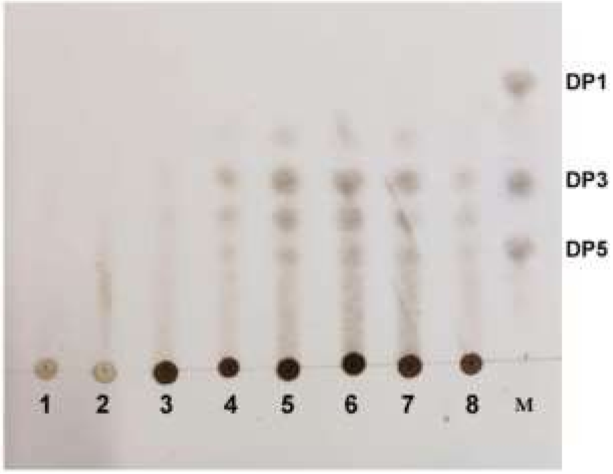

Figure S4. TLC detection results of AOS. Lane 1-8, enzymolysis samples with enzymolysis time of 0, 2, 6, 12, 24, 36, 48 and 60 h; Lane M, the purified monomeric sugar, trimer and pentamer standards.

Table S1. Characteristics of the alginate lyases identified in the genome of strain HB172198^T.

Items	ORF02660	ORF02668	ORF00773	ORF03334
Accession No.	QCT03372.1	QCT03380.1	QCT01494.1	QCT04045.1
Predict function	Alginate lyase	Alginate lyase	Alginate lyase	Alginate lyase
PL family	PL 15	PL 7	PL 38	PL 38
Length of amino acid sequence (aa)	772	312	1013	1611
Molecular weight (Dal)	89150.79	33259.02	113748.01	180048.79
Signal P	No	Yes	Yes	No
PI	5.34	9.28	5.03	4.83

Table S2. The ability of carbohydrates-utilization by strain HB172198^T

Carbohydrates	Culture OD600
sodium alginate	1.152
carrageenan	0.896
agar	0.541
colloidal chitin	0.966
starch	0.621
cellulose	0.326
hemicellulose	0.344
xylan	0.699
xanthan	0.874
sucrose	1.002
lactose	0.265
maltose	0.698
glucose	0.987
rhamnose	0.684
fructose	0.867
xylose	0.984
arabinose	0.548
